# Childhood road traffic injuries in Canada – a provincial comparison of transport injury rates over time

**DOI:** 10.1186/s12889-018-6269-9

**Published:** 2018-12-06

**Authors:** Liraz Fridman, Jessica L. Fraser-Thomas, Ian Pike, Alison K. Macpherson

**Affiliations:** 1 School of Kinesiology and Health Science, York University, 4700 Keele Street, Toronto, ON M3J 1P3 Canada; 2 Department of Pediatrics, University of British Columbia, Vancouver, BC Canada

**Keywords:** Injury prevention, Policy, Epidemiology, Motor vehicle collisions, Child

## Abstract

**Background:**

In Canada, road traffic injuries are the leading cause of death among children and youth ≤19. Across the country, there is variability in road traffic injury prevention policies and legislation. Our objective was to compare pediatric road traffic related injury hospitalization and death rates across Canadian provinces.

**Methods:**

Population-based hospitalization and death rates per 100,000 were analyzed using data from the Discharge Abstract Database and provincial coroner’s reports. Road traffic related injuries sustained by children and youth ≤19 years were analyzed by province and cause between 2006 and 2012.

**Results:**

The overall transport-related injury morbidity rate for children in Canada was 70.91 per 100,000 population between 2006 and 2012. The Canadian population-based injury hospitalization rates from all transport-related causes significantly decreased from 85.51 to 58.77 per 100,000 (− 4.42; *p* < 0.01; − 5.42; − 3.41) during the study period. Saskatchewan had the highest overall transport related morbidity rate (135.69 per 100,000), and Ontario had the lowest (47.12 per 100,000). Similar trends were observed for mortality rates in Canada.

**Conclusions:**

Transport-related injuries among children and youth have significantly decreased in Canada from 2006 to 2012; however the rates vary by province and cause.

**Electronic supplementary material:**

The online version of this article (10.1186/s12889-018-6269-9) contains supplementary material, which is available to authorized users.

## Background

Road traffic collisions are the leading cause of injury death among Canadian children and youth (ages 1–19 years) [1]. The total economic burden to Canadians in 2010 from transport incidents for all ages was $4.2 billion [2]. Male and female adolescents aged 15–19 had the highest rates of transport-related death, 17.04 and 8.00 per 100,000 respectively, compared to younger children [2]. Motor-vehicle collisions (MVCs) accounted for 50% of all transport-related injury costs, followed by pedal cyclists (14%) and pedestrians (11%) [2]. Global comparisons demonstrate that Canada’s childhood mortality rates are similar to most European countries except for Sweden, Italy, and Finland, which are much lower [3]. Yanchar et al. (2012) reported that if Canada’s injury rate was comparable to that of Sweden in 1991–1995 then during this time period, 1233 children would not have died; 23,000–50,000 would not have been hospitalized for an injury; and, more than 250,000 would not have visited an emergency department [4]. Canada ranked 22 out of 30 Organisation for Economic Cooperation and Development (OECD) nations for health and safety in 2009 [5]. According to the Global Burden of Disease Tool, the death rate from road traffic injuries has decreased globally over time for children aged 15–14 years. In 2006, the death rate from occupant, cyclist, and pedestrian related road traffic injuries decreased from 2.14 to 1.92 per 100,000, 0.42 to 0.36 per 100,000 and 3.32 to 2.86 per 100,000, respectively [6]. The burden of injury in Canada has been outlined in many studies, however a comparison of Canadian provinces in terms of injury hospitalization, death rates, and policies related to road traffic injury has not yet been reported [7, 8]. There is considerable variation among road traffic injury prevention policies and legislation across Canada. Evidence-based injury prevention policies such as those related to motor vehicle occupant safety (graduated driver’s licensing [GDL]; booster seat legislation), pedestrian safety, and cyclist safety (helmet legislation) are effective at reducing child and youth injuries, but these policies differ among provinces [9]. In 2015, Macpherson et al. evaluated a number of pediatric injury prevention policies across Canadian provinces. Using a snowball sampling technique for each province, the researchers compared key informants’ perceptions of the quality of three evidence-based injury prevention policies (GDL, booster seat legislation, helmet legislation) [9]. The authors reported that experts rated injury prevention policies that aligned with best practice, such as GDL, higher than policies that did not align with best practice (such as bicycle helmet legislation that did not target all ages). Key informants were also likely to rate public awareness and enforcement higher for the policies that followed best-practice guidelines. Despite evidence that shows that certain injury prevention policies such as GDL, booster seat legislation, and helmet legislation are effective in reducing pediatric injuries, there is still a lack of harmonization across provinces in adopting and enforcing these policies and legislation [3]. Previous studies have examined the rates of pediatric transport-related hospitalizations and deaths over time nationally, but to the authors knowledge, no study has compared pediatric transport-related injury rates provincially in Canada. Using the same methodology as Fridman et al. (2018) the authors wanted to extend their work on interprovincial injury comparisons to road traffic-related causes [7].

Our objective in this study was to compare pediatric road traffic-related injury hospitalization and death rates across Canadian provinces.

## Methods

### Data collection

Using data from the Discharge Abstract Database (DAD), provided by the Canadian Institute for Health Information (CIHI), we conducted a retrospective analysis of population-based injury hospitalizations from road traffic incidents. CIHI originally developed the DAD in 1963 [10]. This database collects information on hospital discharges including deaths, sign-outs, and transfers. Data from the DAD is also used to populate other CIHI databases such as the hospital mortality database and the hospital mental health database [10].

Data is collected from all provinces and territories except for Quebec (QC), who are not required to report this data. The data is available for fiscal years 1979–1980, and 1994 onwards. The DAD contains demographic, administrative, and clinical data for hospital inpatient discharges and day surgery interventions. This data is collected primarily from diagnostic coding that relies on a review of the patient’s chart to produce important health information such as health history and current diagnoses. This data is collected by health professionals who assign diagnostic codes using the International Classification of Diseases and Related Health Problems (currently coded using ICD-10-CA). External causes of injury were grouped based on the ICD-10 codes. All unintentional transport injury codes V01-V99 were analyzed. Children and youth (0–19 years) who were hospitalized after sustaining a road traffic-related injury between January 1, 2006 and December 31, 2012 in all provinces, excluding QC, were included in this study.

We obtained the number of childhood deaths from chief coroners or medical examiners in each province. This provincial coronial data was used to analyze the death rate among children and youth (0–19 years) who died after sustaining a transport-related injury between January 1, 2006 and December 31, 2012 in all provinces. Data analyses were conducted at the Research Data Centres at York University using SPSS version 24. Ethics was obtained from York University.

### Study variables

We examined the number of road traffic-related injury hospitalization and deaths in Canada between 2006 and 2012 as our primary outcome measure. Variables including; cause of injury, year, and province of residence were analyzed, where applicable, and confidence intervals for trends over time were reported.

### Statistical analyses

Population-based rates per 100,000 were calculated for both hospitalization and death data. Hospitalization data was also analyzed as an average annual incidence rate and parameter estimates with 95% confidence intervals are presented. Mortality rates were calculated for road traffic fatality data.

## Results

### Unintentional transport-related injury rates in Canada

Between 2006 and 2012, the population-based hospitalization rate for transport-related injuries was 70.91 per 100,000 for Canadian children and youth. Over the seven-year study period, transport-related injuries decreased significantly from 85.51 to 58.77 per 100,000 (− 4.42; *p* < 0.01; − 5.42; − 3.41). Saskatchewan (SK) had the highest average transport-related morbidity rate (135.69 per 100,000) compared to the Canadian average, and Ontario (ON) had the lowest (47.12 per 100,000). SK population-based hospitalization rate decreased significantly (− 6.38; p < 0.01; − 10.62; − 2.14) over time. All nine Canadian provinces included in the analysis showed a decrease in transport-related injury morbidity rates between 2006 and 2012 (see Table 1).

**Table 1:** Population Based Injury Hospitalization Rate per 100,000 from all transport-related causes by Canadian Province (2006–2012) among children and youth, 0–19 years

	2006	2007	2008	2009	2010	2011	2012	Average Annual Rate	Parameter Estimate	(95% CI)
NB	153.24	115.21	104.85	103.27	88.21	84.31	85.31	105.34	− 10.1	(− 15.77; −4.38)
MB	118.11	95.69	93.22	84.90	87.03	83.77	67.45	89.93	−6.50	(−9.81; −3.19)
BC	103.08	99.76	80.82	81.08	71.37	68.82	60.76	80.84	−7.08	(−9.09; −5.07)
ON	56.16	54.33	48.98	43.52	43.01	45.05	38.65	47.12	−2.75	(−3.96; − 1.54)
PEI	136.20	102.18	93.54	136.1	78.97	100.1	97.87	106.53	−4.78	(−14.92; 5.37)
AB	114.57	109.27	105.47	98.58	87.23	86.71	83.42	97.65	−5.60	(−6.89; −4.31)
NL	138.22	122.16	148.51	96.72	87.53	107.92	108.08	115.77	−6.42	(−15.49; 2.64)
SK	146.80	148.59	154.56	143.33	119.87	119.67	118.1	135.69	−6.38	(−10.62; −2.14)
NS	81.18	78.27	69.56	69.59	72.67	81.10	77.74	75.73	−0.06	(−3.67; 2.63)
Canada^**^	85.51	80.63	74.47	69.01	63.65	64.34	58.77	70.91	−4.42	(−5.42; −3.41)

A positive change indicates an annual increase over time; a negative change indicates a decrease over time. Confidence intervals that do not cross zero are

statistically significant. NL and PEI could not be reported by this subcause since cell sizes were < 5

^**^Excludes Quebec and territories

The mortality rate from all road traffic-related injuries in Canada was 4.50 deaths per 100,000 children/youth between 2006 and 2012. Compared to the Canadian average, the highest mortality rate was 10.99 per 100,000 population in SK and the lowest rate was 3.09 per 100,000 in ON (see Table 2).

**Table 2:** Population Based Mortality Rate per 100,000 (2006–2012) from all transport related injuries among children and youth, 0–19 years

Province	All Road Traffic Injuries
ON	3.09
NL	4.19
BC	4.45
AB	5.50
NS	5.60
MB	6.41
PEI	6.90
NB	10.38
SK	10.99
CAN	4.50

### Provincial comparisons of occupant-related injury hospitalization

The average rate of childhood occupant-related injury hospitalization in Canada was 22.05 per 100,000 population. Between 2006 and 2012, the population-based injury morbidity rate decreased significantly from 28.64 to 16.97 per 100,000 (− 1.93; *p* < 0.01; − 2.41; − 1.46) for children who were occupants in a MVC (see Table 3). Prince Edward Island (PEI) had the highest occupant-related population-based morbidity rate (55.64 per 100,000) when compared to the Canadian average, and ON had the lowest rate (13.81 per 100,000). All nine Canadian provinces analyzed showed a decrease in occupant-related injury morbidity rates between 2006 and 2012.

**Table 3:** Occupant-related injury hospitalization rate per 100,000 between 2006 and 2012 by Province among children and youth, 0–19 years

	2006	2007	2008	2009	2010	2011	2012	Average Annual Rate	Parameter Estimate	(95% CI)
PEI	94.75	63.11	39.23	51.41	54.67	51.55	33.64	55.64	− 6.82	(− 13.93; 0.29)
BC	29.48	28.81	24.91	20.99	19.55	19.35	12.55	22.24	−2.68	(−3.45; −1.91)
NB	47.70	39.81	29.26	36.27	32.92	28.94	22.92	34.12	−3.30	(−5.40; −1.20)
MB	42.43	32.95	30.65	33.64	33.81	27.72	21.66	31.80	−2.50	(−4.30; −0.68)
ON	19.24	16.74	14.72	11.56	12.16	11.47	10.69	13.81	−1.38	(−2.01; −0.76)
AB	41.69	36.94	33.12	28.21	27.79	24.83	27.67	31.36	−2.56	(−3.87; −1.24)
NL	32.10	38.91	29.15	26.46	21.19	37.21	23.49	29.83	−1.32	(−4.55; 1.89)
NS	28.32	34.57	24.32	26.16	25.56	32.65	22.80	27.81	−0.68	(−2.87; 1.50)
SK	54.16	62.35	67.83	56.37	42.16	41.96	46.81	53.01	−3.16	(−7.03; 0.71)
Canada**	28.64	26.46	23.41	20.53	19.55	18.82	16.97	22.05	−1.93	(− 2.41; − 1.46)

Apositive change indicates an annual increase over time; a negative change indicates a decrease over time. Confidence intervals that do not cross zero are

statistically significant

**Excludes Quebec and territories

### Provincial comparisons of cyclist-related injuries

The average rate of childhood cyclist-related injury hospitalization in Canada was 17.58 per 100,000 population. Between 2006 and 2012, the population-based injury morbidity rate decreased significantly from 21.87 to 14.30 per 100,000 (− 1.16; *p* < 0.01; − 1.61; − 0.71) for child cyclists (see Table 4). NB had the highest cyclist-related population-based morbidity rate of any province (27.87 per 100,000) when compared to the Canadian average, and ON had the lowest (13.72 per 100,000). Eight of nine provinces analyzed showed a decrease in cyclist-related injury morbidity rates from 2006 to 2012.

**Table 4:** Cyclist-related injury hospitalization rate per 100,000 between 2006 and 2012 by Province among children and youth, 0–19 years

	2006	2007	2008	2009	2010	2011	2012	Average Annual Rate	Parameter Estimate	(95% CI)
NB	44.72	32.57	32.92	30.12	18.01	17.62	17.82	27.87	− 4.48	(−6.39; − 2.58)
MB	21.53	17.74	13.27	14.46	15.03	14.64	12.07	15.52	−1.17	(−2.18; − 0.16)
NL	36.56	24.43	20.95	24.64	19.35	18.61	20.68	23.68	−2.17	(−4.30; −0.05)
BC	34.22	31.91	20.98	24.30	22.24	20.39	19.80	24.84	−2.32	(−3.92; −0.73)
NS	28.32	15.85	22.38	15.79	16.04	13.77	16.58	18.49	−1.63	(−3.61; 0.35)
ON	16.65	15.81	13.57	12.65	12.22	14.34	10.72	13.72	−0.79	(−1.42; −0.16)
SK	25.03	22.77	19.64	19.90	17.23	20.80	18.36	20.51	−0.94	(−1.85; − 0.04)
AB	18.44	21.12	22.62	20.86	17.70	18.78	17.96	19.63	−0.39	(−1.29; 0.50)
PEI	*	*	18.10	39.32	*	*	*	N/A	N/A	N/A
Canada**	21.87	20.19	17.67	17.20	15.45	16.39	14.30	17.58	−1.16	(− 1.61; − 0.71)

A positive change indicates an annual increase over time; a negative change indicates a decrease over time. Confidence intervals that do not cross zero are

statistically significant. PEI could not be reported by this subcause since cell sizes were < 5

N/A not applicable

*Cell size < 5

**Excludes Quebec and territories

### Provincial comparisons of pedestrian-related injuries

The average rate of childhood pedestrian-related injury hospitalization in Canada was 7.51 per 100,000 population. Between 2006 and 2012, the population-based injury morbidity rate decreased significantly from 8.29 to 6.29 per 100,000 (− 0.29; *p* < 0.01; − 0.49;-0.08) for child pedestrians (see Table 5). SK had the highest pedestrian-related population-based morbidity rate (13.31 per 100,000) when compared to the Canadian average, and PEI had the lowest rate (3.02 per 100,000). Eight of nine provinces analyzed showed a decrease in pedestrian-related injury morbidity rates from 2006 to 2012.

**Table 5:** Pedestrian-related injury hospitalization rate per 100,000 between 2006 and 2012 by Province among children and youth, 0–19 years

	2006	2007	2008	2009	2010	2011	2012	Average Annual Rate	Parameter Estimate	(95% CI)
PEI	*	*	*	*	*	*	*	N/A	N/A	N/A
AB	9.84	8.07	6.89	8.97	6.87	9.13	5.64	7.90	−0.38	(−1.05; 0.30)
SK	13.82	14.19	16.31	13.26	13.56	13.13	9.00	13.31	−0.70	(−1.54; 0.16)
BC	12.37	13.12	9.41	9.31	8.69	9.42	8.09	10.06	−0.75	(−1.29; − 0.21)
NB	4.77	4.22	6.71	8.61	4.35	6.92	3.18	5.54	−0.06	(−1.08; 0.96)
NL	11.59	9.95	18.22	9.12	8.29	6.51	8.46	10.33	−0.94	(−2.66; 0.79)
ON	5.66	6.51	5.77	5.78	5.34	6.25	5.09	5.77	−0.10	(−0.33; 0.14)
MB	13.62	12.04	12.01	10.38	7.51	9.03	12.38	10.99	−0.51	(−1.48; 0.46)
NS	7.08	3.36	5.35	5.92	10.53	5.61	7.77	6.49	0.42	(−0.68; 1.52)
Canada**	8.29	8.32	7.55	7.61	6.89	7.64	6.29	7.51	−0.29	(−0.49; − 0.08)

A positive change indicates an annual increase over time; a negative change indicates a decrease over time. Confidence intervals that do not cross zero are

statistically significant. PEI could not be reported by this subcause since cell sizes were < 5

N/A not applicable

*Cell size < 5

**Excludes Quebec and territories

## Discussion

All cause transport-related injury hospitalization rates significantly decreased among children and youth over the 7-year study period. However, rates of hospitalization resulting from road traffic incidents differed among provinces. During the study period, Saskatchewan experienced an injury hospitalization rate almost double that of the Canadian average. Transport-related injury prevention policies targeted at occupants, cyclists, and pedestrians such as GDL, booster seat legislation, and helmet legislation, vary substantially among and within provinces.

One policy that may have influenced the decrease in motor vehicle-related injury hospitalization rates over time is GDL which requires novice drivers to advance through a number of learning phases where they are supervised in lower-risk conditions until they gain more knowledge and experience on the roads [11]. In the United States, GDL programs that combined a mandatory waiting period of more than 3-months between stages; a nighttime driving restriction; and, greater than 30-h of supervised driving and/or passenger restrictions, were associated with a 16–21% reduction in fatal crashes among teen drivers [12]. All provinces in Canada require new drivers to pass a learner/novice phase. The majority of provinces (BC, AB, ON, NS, NB, NL) require 2 levels/phases before being a fully licensed driver. However, some provinces (SK, MB, PEI) also have a third stage that must be successfully passed before being permitted to drive without restrictions. The age at which drivers can obtain a learner’s permit varies by province, with youth as young as 14-years being able to apply for a learner’s permit in Alberta. In the majority of provinces, the minimum age is 16-years old. In SK, MB and PEI if the driver is 15-years of age and enrolled in the high school driver education program they are eligible to receive a learner’s permit. All provinces require new drivers to have a supervisor in the car at the first/novice stage. MB, ON, PEI, NL require the supervisor to be fully licensed and have blood alcohol concentration restrictions, whereas BC, AB, SK, NS, NB only have varying age and licensure requirements. All provinces require novice drivers to have a zero blood alcohol content (BAC) level during their level/stage one driver training. In addition to differences between the number of phases, minimum age and supervisory requirements, provinces also differ with regard to mandatory driver education programs, and nighttime and passenger restrictions (see Additional file 1) [13]. A Cochrane Systematic Review concluded that stronger GDL programs (i.e. programs that involve more restrictions for novice drivers) appear to result in a greater reduction in mortality from motor vehicle crashes among young drivers [14]. Our study found that ON had the lowest mortality rate from road traffic-related causes. ON was also one of the first provinces to introduce GDL and restricts novice drivers from being on the roads between 12:00–5:00 AM. These findings suggest that GDL may be an important injury prevention policy that has the potential to reduce injury-related deaths.

Occupant-related injuries decreased in all provinces during the study period. This may be due to effective booster seat legislation implementation across the country. In a number of studies, child motor vehicle restraints, including rear-facing car seats, forward-facing car seats, and booster seats were shown to significantly reduce the risk of severe injury and death among children who are involved in a MVC [4]. In Canada, there is variability in the specifics and requirements of booster seat legislation. Some provinces include only age, weight, and height restrictions, whereas other provinces include additional components such as driver responsibility, non-compliance penalties, and public education and incentive programs. Snowdon et al. (2009) performed the first Canadian study that evaluated the effectiveness of booster seat legislation on restraint use among children aged 4–8 years old. They found that in provinces with legislation, 91.9% of children were restrained and of those 24.6% used a booster seat specifically. Conversely, 84.4% of children in provinces without legislation were restrained, and of those, only 16.6% used a booster seat [15]. In all provinces with booster seat legislation, children must ride in a booster seat until they are a minimum of 4 ft, 9 in. (145 cm) tall, or a minimum of 9-years of age. In addition, SK, MB, ON, PEI, NB, and NL also have weight recommendations, requiring a child to have reached a body weight of between 18 and 36 kg before graduating from a booster seat. Every province except NL has penalties for drivers who do not comply with the use of an appropriate booster seat to restrain child passengers (see Additional file 1). These penalties have been in place since 2009 for every province except for MB (enacted penalties in 2013) and SK (enacted penalties in 2014). At the time of writing, AB is still the only province that does not have booster seat legislation in place.

Provinces in Canada also varied in the requirements and penalties related to driver responsibility, public education, and incentive programs for booster seats [16]. Our study found that ON and BC were the provinces with the lowest average annual rates in occupant-related hospitalization over time. BC’s booster seat legislation has been in place since 1985 but was updated to reflect evidence-based best practice in 2008. Brubacher et al. (2016) found that the updated booster seat law was associated with a 10.8% (95% CI 2.7 to 18.9%) reduction in the monthly rate of injuries in four- to eight-year-old children [17]. In addition to age and height requirements there are also stipulations on public education, incentive programs, non-compliance penalites, and driver responsibility in BC. These findings suggest that more comprehensive booster seat legislation may play a role in reducing occupant-related hospitalizations. This study provides evidence to support booster seat legislation and educational messages and materials that are consistent across provinces.

Cycling-related hospitalization rates decreased in Canada over the study period in all provinces with the exception of PEI where cell sizes were too small to report. Legislation requiring citizens to wear a bicycle helmet while cycling varies among provinces. Some provincial laws apply to Canadians of all ages whereas others only apply to children and youth who are less than 18-years of age. In addition, some provinces have enacted legislation that applies to all-wheeled activities including skates, skateboards, and push-scooters. Other differences include where these laws are enforced (all roads vs. public roads) and the extent to which individuals are penalized (variation in monetary fines) [9]. In Canada, three provinces (AB, MB, ON) do not have helmet legislation that applies to all ages, but instead applies to children/youth less than 18-years of age, and at the time of writing there is no provincial law requiring the use of bicycle helmets in Saskatchewan [18] . BC and NS also have legislation that requires individuals participating in all-wheeled activities including skates, skateboards, and push-scooters to wear a helmet (see Additional file 1). Penalties for not complying with bicycle helmet use also vary by province, with the smallest fine of $21 in NB and the greatest fine of up to $100 in BC, PEI, and NL [19]. Our study found contradictory results to some previous work on cycling related injuries and bicycle helmet legislation. NB had the highest average annual rate in cycling-related injuries over time and has the lowest fine of any province, however NB was also one of the first provinces to implement bicycle helmet legislation despite not including all ages. Studies have previously suggested that bicycle helmet legislation that targets all ages achieves higher levels of helmet use than laws that apply to children [20–22]. The two provinces with all ages helmet legislation (BC & NS) demonstrated a significant decrease in cycling related hospitalizations in our study however decreases were larger in other provinces such as MB that did not have all ages legislation. This study did not examine head-related hospitalizations specifically, but rather the rates of hospitalizations for all cycling-related injuries. This may account for the difference between our findings and those of previous studies in this area. This variability may also be attributed to other factors such as the cycling environment. Given the many different factors that may influence injury rates, we need to consider harmonizing provincial policies so we can continue to advance research and best practices to reduce childhood injury morbidity and mortality rates.

Pedestrian safety laws differ at the municipal level in Canada. Policies related to pedestrian safety are multifactorial and can involve designing safe routes for children to walk to school including sidewalk design, traffic calming, on-street parking limits, having adequate numbers of trained crossing guards, and escort programs for young children [20]. Active transportation studies have examined the relationship between observed walking to school and child pedestrian collisions; these studies suggest that modification to the built environment may promote both walking to school and increased pedestrian safety [23]. Our study found that the only province with a significant reduction in pedestrian-related injuries was BC. Pedestrian safety laws in BC include speed limit reductions in residential zones of 50 km/h and 30 km/h in school zones. Higher speed limits are one factor that has been associated with increased risk of impact with vulnerable road users such as cyclists and pedestrians [24]. Implementation of standardized, evidence-based policies and strategies such as reduced speed limits in residential and schools zones across provinces may be one effective way to reduce road traffic injuries.

### Strengths

Previous studies that examine pediatric injury data over time focus on national rates in Canada. This study was novel in that we compared differences in population-based rates of hospitalization and death from a number of road traffic-related causes among Canadian provinces over a 7-year study period. We also related our findings to provincial prevention policy/legislation, where applicable.

### Limitations

Due to differences in reporting standards at a provincial and national level, we were unable to report mortality data by province and cause, by year. However, mean provincial mortality rates for the 7-year study period were computed and compared. At the time of writing, the most recent data that was available to us from CIHI and the coroner’s offices was from 2012, as such changes to the rates of pediatric injury hospitalization and death have likely changed since then. As a multitude of factors affect injury outcomes beyond the presence of policy and legislation alone, this study does not demonstrate a causal relationship between the presence of policy and injury outcomes. Each province also differs on a number of environmental features such as weather, topography, and rural/urban dispersion therefore exposure to injury will differ among provinces which affects the observed change in rate over time. The associative relationships however, coupled with the comparison of injury outcomes in provinces with and without ‘best practice’ policies, suggests a protective effect of well-developed road traffic-safety legislation. Further examination of these relationships is warranted.

## Conclusions

While transport-related injuries among Canadian children and youth continue a downward trend, inconsistences between road traffic safety policies across the country persist. This study highlights the need for evidence-based policies targeted towards occupant, cyclist, and pedestrian safety such as GDL, bicycle helmet legislation, pedestrian safety laws, and booster seat legislation to follow best practice guidelines and be standardized across Canada.

## Additional file

Additional file 1: **Table S1** Graduated Driver’s Licensing by Province. **Table S2** Booster Seat Legislation by Province, **Table S3** Bicycle Helmet Legislation by Province. (DOCX 16 kb)

## Abbreviations

AB: Alberta
BC: British Columbia
GDL: Graduated Driver’s Licensning
MB: Manitoba
MVC: Motor Vehicle Collisions
NB: New Brunswick
NL: Newfoundland & Labrador
NS: Nova Scotia
ON: Ontario
PEI: Prince Edward Island
PMVC: Pedestrian Motor Vehicle Collision
QC: Quebec
SK: Saskatchewan
