# Supplementary material for: Childhood road traffic injuries in Canada – a provincial comparison of transport injury rates over time
Source: BMC Public Health. 2018 Dec 6;18:1348. doi: 10.1186/s12889-018-6269-9 (PMC6282394; doi:10.1186/s12889-018-6269-9)
Supplement: Supplementary file 1 — Table S1 Graduated Driver’s Licensing by Province. Table S2 Booster Seat Legislation by Province, Table S3 Bicycle Helmet Legislation by Province. (DOCX 16 kb) [file 12889_2018_6269_MOESM1_ESM.docx]

**Additional File 1**

**Table 1: Graduated Driver’s Licensing by Province**

|  | Year of Implementation | Supervisory Driver (Yes/No),  Minimum Age | Driver education/training | Blood Alcohol Content (BAC) | Restrictions:  Night (Y/N)  Passengers (Y/N) | Sign-on vehicle |
| --- | --- | --- | --- | --- | --- | --- |
| **PROVINCE** | | | | | | |
| British Columbia | 1998 | Yes (25 years or older with a valid Class 1-5 driver’s license)  16 years | Voluntary | Zero | Yes: no driving between 12 AM – 5 AM  Passengers: limit of 2 | Mandatory |
| Alberta | 2003 | Yes (18-year-old; fully licensed)  14 years | Voluntary | Zero | Yes: no driving between 12 AM – 5 AM  Passengers: limited to number of working seatbelts | None |
| Saskatchewan | 2005 | Yes: occupies the front passenger seat  16 years or 15 years (if enrolled in the high school education program) | Mandatory for all new drivers | Zero | Only immediate family permitted in vehicle between midnight and 5 a.m.  Passengers: limited to number of seatbelts | None |
| Manitoba | 2003 | Yes: Fully licensed for at least 3 years, 0 BAC  16 years or 15 years (if enrolled in the high school education program) | Voluntary | Zero | No night time restrictions  Limited to 1 supervising driver in the front seat, and number of working seatbelts | None |
| Ontario | 1994 | Yes: Fully licensed, with at least 4 years driving experience, BAC<.05, seated in front seat  16 years | Voluntary | Zero | Yes: no driving between 12 AM – 5 AM  Must have supervisor at all times; other passengers limited to number of seatbelts | None |
| Quebec | 1997 | Yes: Fully licensed for 2 years, BAC<=.08, seated in front seat  16 years | Mandatory | Zero | None | None |
| Nova Scotia | 1994 | Yes: Experienced driver with at least a Class 5 license  16 years | Mandatory  Long course: 25 hours in class & 10 hours in car  Short course: 6 hours in class | Zero | May drive after midnight with supervisor  No passengers except supervisor | None |
| Prince Edward Island | 2000 | Yes: Has valid license for at least 4 years for same class of vehicle, BAC<.05  16 years or 15 years (if enrolled in the high school education program) | Voluntary | Zero | Refrain from driving between 1 a.m. and 5 a.m. for drivers under 21 years  No passengers, except supervisor or family members | Mandatory |
| New Brunswick | 1996 | Fully licensed, seated in front seat  16 years | Voluntary | Zero | Yes: no driving between 12 AM – 5 AM  No passengers except supervisor | None |
| Newfoundland and Labrador | 1999 | Four years of driving experience, BAC = 0  16 years | Voluntary | Zero | Yes: no driving between 12 AM – 5 AM  No passengers except supervisor (except for parents/guardians if driver is enrolled in driver education and accompanied by a licensed instructor) | Mandatory |

**Table 2: Booster Seat Legislation by Province**

|  | **Year of Implementation** | **Age** | **Height/Weight** | **Public Education** | **Incentive Program** | **Non-Compliance Penalties** | **Driver Responsibility** |
| --- | --- | --- | --- | --- | --- | --- | --- |
| **Province** |  | | | | | | |
| **BC** | 2008 | 9 years old | 4 feet 9 inches (145 cm); no weight restrictions | YES | YES | YES | YES |
| **AB** | **NO PROVINCIAL LEGISLATION** | | | | | | |
| **SK** | 2014 | 7 years old | 4 feet 9 inches (145 cm); 80 lbs (36 kg) | YES | YES (since 2014) | YES (since 2014) | NO |
| **MB** | 2012 | 9 years old | 4 feet 9 inches (145 cm); 80 lbs (36 kg) | YES | NO | YES (since 2013) | YES |
| **ON** | 2005 | 8 years old | 4 feet 9 inches (145 cm); 80 lbs (36 kg) | YES | YES | YES | YES |
| **QC** | 2002 | No age restrictions | 25 inches (63 cm); no weight restrictions | YES | NO | YES | NO |
| **NS** | 2007 | 9 years old | 4 feet 9 inches (145 cm); no weight restrictions | YES | YES | YES | YES |
| **PEI** | 2008 | 9 years old | 4 feet 9 inches (145 cm); 40 lbs (18 kg) | YES | YES | YES | YES |
| **NB** | 2008 | 9 years old | 4 feet 9 inches (145 cm); 80 lbs (36 kg) | YES | NO | YES | YES |
| **NWFL** | 2008 | 4 and 8 years old | 4 feet 9 inches (145 cm); between 40 lbs (18 kg) and 80 lbs (36 kg) | NO | NO | NO | NO |

**Table 3: Bicycle Helmet Legislation by Province**

|  | Age | Effective Date | Penalty |
| --- | --- | --- | --- |
| **PROVINCE** | | | |
| British Columbia | Applies to all ages | September 3, 1996  2003: updated to include helmet use for all wheeled activities including skates, skateboards, and push-scooters | Fine up to: $100 |
| Alberta | Applies only to those <18 years of age | May 1, 2002 | Fine: $69 |
| Saskatchewan | **NO PROVINCIAL LAW** | | |
| Manitoba | Applies only to those <18 years of age | May 1 2013 | Fine up to: $50 |
| Ontario | Applies only to those <18 years of age | October 1, 1995 | Fine: $60 |
| Quebec | **NO PROVINCIAL LAW** | | |
| Nova Scotia | Applies to all ages | July 1, 1997  2007: updated to include helmet use for all wheeled activities including skates, skateboards, and push-scooters | Fine minimum: $25 |
| Prince Edward Island | Applies to all ages | July 5, 2003 | Fine up to: $100 |
| New Brunswick | Applies to all ages | December 15, 1995 | Fine: $21 |
| Newfoundland and Labrador | Apples to all ages | April 1, 2015 | Fine up to: $100 |
